# Studying Under Stress: The Effect of COVID-19 Psychological Distress on Academic Challenges and Performance of Post-Secondary Students

**DOI:** 10.1177/15210251221104245

**Published:** 2022-06-05

**Authors:** Paweena Sukhawathanakul, Allyson Hadwin, Ramin Rostampour, Michelle Bahena Olivares, Kate Shostak

**Affiliations:** 8205 University of Victoria, Canada

**Keywords:** post-secondary students, COVID-19, academic performance

## Abstract

The COVID-19 pandemic introduced significant disruptions in the learning environment for many post-secondary students. While emerging evidence suggest mental health has declined during the pandemic, little is known about how the pandemic has affected students academically. This study investigates how COVID-19 psychological distress impacts academic performance among a Canadian sample of post-secondary students (n = 496). Path analysis findings suggest that greater levels of COVID-19 distress was associated with lower self-reported predicted GPA. Metacognitive, motivational, and social and emotional challenges emerged as the most salient challenge areas that fully mediated the relationship between COVID-19 psychological distress and self-reported predicted GPA. Specifically, COVID-19 distress predicted greater levels of metacognitive and motivational challenges which, in turn, predicted lower self-reported GPA. Similarly, greater levels of COVID-19 distress predicted more social and emotional challenges but these challenges were associated to higher perceived GPA. Findings warrant future research to help students manage and cope with academic challenges that may be exacerbated under stressful conditions.

The COVID-19 pandemic has created a significant shift in daily behaviors worldwide with many adhering to social distancing guidelines by primarily working and studying from home. In response to ongoing pandemic restrictions, most Canadian post-secondary institutions transitioned to full-time online learning in order to ensure that students remain safe while completing their studies. However, this shift in students’ learning environment may introduce challenges that impede their academic engagement. Psychological distress, feelings of isolation, technology-related issues (e.g., internet connectivity), and limited access to research resources and faculty pose challenges to students’ motivation to learn and to their academic performance (e.g., grade point average; GPA). Such disruption to the student experience can ultimately contribute to an increase in dropout rates. In addition to reporting disruptions to their learning such as not being able to secure courses or completing their degrees on time (Doreleyers & Knighton, 2020), the pandemic has also exacerbated the economic burden of Canadian students. Post-secondary students report considerable financial strain with over 70% of students using up their savings or having to take on more student debt during the early stages of the pandemic (Statistics Canada, 2020). In addition to increasing worries about their income, post-secondary students report heightened worries related to health uncertainties especially if they were currently living with immunocompromised individuals (Cao et al., 2020). These stressors related to economic hardship and uncertainty likely contribute to the rise in anxiety symptoms reported by post-secondary students during the pandemic (Dodd et al., 2021; Patterson et al., 2021).

Increasing worries related to income, housing, social and health uncertainties can exacerbate academic challenges which may be compounded under pandemic restrictions as students transition to remote online learning (Cao et al., 2020). While emerging evidence suggest that the increased amount of stress introduced by the pandemic may be impacting students’ academic performance and engagement (e.g., Meo et al., 2020), little is known about the nature of specific academic challenges that may be impeding students’ academic performance. In this study, we investigate how psychological distress related to the pandemic impacts post-secondary students’ academic performance (measured as self-reported predicted GPA). We also examine how psychological distress related to the pandemic predicts different types of academic challenges (cognitive, motivational, socio-emotional, metacognitive, and behavioral) and test whether these challenges differentially mediate the relationship between COVID-19 psychological distress and academic performance.

## COVID-19 and Post-Secondary Students

While distance learning is not a novel approach in the post-secondary setting, the abrupt shift from face-to-face learning to remote online learning during the COVID-19 outbreak has globally introduced unique challenges to students who are unfamiliar with self-regulated learning in an online environment. In an Algerian sample of post-secondary students, Blizak et al. (2020) found that the majority of students in the study reported negative perceptions towards to the shift to online learning citing a range of technical (e.g., internet outages, lack of personal computers), psychological (e.g., stress, lack of time), and pedagogical obstacles (e.g., lack of community with teachers). Similarly, in a sample of Canadian undergraduate students, Daniels et al. (2021) found that the shift to online learning during the pandemic decreased students’ achievement goals, engagement and perceptions of success. In an American longitudinal sample, Fruehwirth et al. (2021) found that increases in depression and anxiety symptoms among post-secondary student during the COVID-19 outbreak was associated with distanced learning and social isolation. In a sample of Australian undergraduates, students who reported more negative learning environments during the pandemic had lower psychological well-being compared to students who were more academically adjusted during the pandemic (Dodd et al., 2021).

While emotional distress resulting from academic difficulties are normative for students, the abrupt shift to remote online learning can exacerbate existing worries related to uncertainties with graduating on schedule and employment prospects (Cao et al., 2020). For students with existing mental health difficulties, the introduction of new academic challenges related to the pandemic coupled with social isolation can further tax already compromised coping capacities. The diathesis-stress model provides an explanation of how environmental triggers such as pandemic conditions can aggravate pre-existing mental health problems and impair everyday functioning. For example, recent research has linked COVID-19 stressors such as social isolation and economic uncertainties with higher traumatic stress in individuals with pre-existing mood disorders (Asmundson et al., 2020). Social isolation and distancing are associated with increases in depressive symptoms and physical health problems (e.g., with sleep, diet) during the pandemic even among individuals without pre-existing mood disorders (Li et al., 2021). Applied in the context of academic performance, accumulating environmental stressors introduced by the pandemic may have spill-over effects on students’ academic performance by introducing more academic challenges related to areas of motivation, cognition, metacognition, behavioral engagement, and socio-emotional functioning.

Emerging evidence suggests that the increased amount of stress introduced by the pandemic may be impacting students’ academic performance and engagement. Students have reported a significant reduction in work performance and time dedicated to studying during the pandemic (Meo et al., 2020), as well as an increase in procrastination and attentional difficulties (Hong et al., 2021). Moreover, students who adapt poorly to learning in an online environment and who are worried about their academic performance have also reported greater psychological distress related to COVID-19 compared to students who reported fewer academic worries (Hasan & Bao, 2020).

However, there is substantial heterogeneity in academic challenges experienced by post-secondary students that differentially moderate academic success yet few studies have accounted for these differences. For example, for some students, cognitive challenges related to difficulties with processing course content and metacognitive challenges related to problem-solving, planning and monitoring of tasks may be more salient (e.g., Mihalca et al., 2017) than motivational challenges (e.g., Bernacki et al., 2015). Some students may have difficulties engaging in behavioral strategies like time management that impair their ability to effectively meet their studying goals while others may have socio-emotional challenges related to the academic experience such as struggles with social relationships, social belonging in the university, and test anxiety (Hadwin et al., 2019).

Self-regulatory learning (SRL) competencies may help to mitigate some of these academic challenges (Zimmerman, 2000), but stressors related to the COVID-19 pandemic can compromise SRL competencies that are necessary to overcome these multifaceted academic challenges that can in turn diminish academic performance. Understanding how psychological distress related to the COVID-19 pandemic impacts the different facets of academic challenges will be important in tailoring academic intervention efforts to better cater to students’ diverse academic needs. The current study investigates associations between COVID-19 psychological distress and different types of academic challenges (cognitive, motivational, socio-emotional, metacognitive, and behavioral), as well as academic performance as indicated by students’ self-reported predicted GPA. We further test whether the different types of academic challenges mediate the relationship between COVID-19 psychological distress and academic performance.

## Methods

### Participants

Participants (N = 496) were enrolled in a Western Canadian post-secondary institution. Participants were recruited from three domains including students who were (a) registered in an academic course on learning strategies for university success (N = 95), (b) included in the psychology research participation pool (N = 369), or (c) accessing a learning strategies library resource available to all students at the university (N = 32). Participants who were registered in the learning strategies academic course were required to participate in the study in order to receive course credit while participants who were drawn from the psychology research participation pool received course credit (that they can allocate to any of their psychology courses) after completing the survey. Participants who were drawn from the online learning strategies resource were entered into a draw for a gift card as compensation for their participation.

#### Procedure

Participants completed an online survey that included measures of COVID-19 psychological distress, self-reported predicted GPA, and academic challenges. After completing the survey students were sent a personalized academic challenge report that allowed students to understand their learning habits and challenges. The study was approved by the Institution's Human Research Ethics Board.

### Measures

COVID-19 psychological distress was measured using the COVID Stress Scales (Taylor et al., 2020) which comprised of (23 items) that yielded 5 sub-dimensions related to (1) danger and contamination fears (e.g., I am worried about catching the virus from handling money or using a debit machine), (2) fears about economic consequences (e.g., “I am worried my financial situation will be affected by COVID-19”), (3) xenophobia (e.g., “I am worried that people around me will infect me with the virus”), (4) compulsive checking and reassurance seeking (e.g., “I sought reassurance from friends and family about COVID-19”), and (5) traumatic stress symptoms related to the pandemic (e.g., Disturbing mental images about the virus popped into my head against my will”). In addition, two items related to feelings of guilt and shame about the pandemic (“I feel guilty for not doing more to prevent COVID-19; “I feel ashamed of my emotional reactions to COVID-19) were also added from the COVID-19 Peritraumatic Distress Index (CPDI; Qiu et al., 2020). The items were rated on a 5-point scale ranging from 0 (not at all) to 4 (extremely) and were summed to create a composite for COVID-19 distress.

Academic performance was measured using self-reported predicted GPA which asked students to report on what overall GPA (average grade) they expected to get this year. Academic challenges were measured using the challenges subscale from the Hadwin et al.'s (2021 ) SRL-academic self-diagnostic tool instrument. Students were asked to rate their level of agreement with 43 statements on a 5-point Likert scale ranging from strongly disagree (−2) to strongly agree (2) which were summed to create a composite. The items tapped 5 subtypes of academic challenges including difficulties related to motivation (e.g., “I am interested in my schoolwork”), behavioral engagement (e.g., “I am managing my time, tasks, or goals”), cognition (e.g., “I am elaborating and connecting with what I learned”), socio-emotional functioning (e.g., “I am taking care of my mental health and well-being”), and metacognition (e.g., “I know how or when to fix strategies/study skills”).

### Analytic Strategy

Path analyzes was used with Mplus v.8 (Muthén & Muthén, 2010) to estimate hypothesized relationships between COVID-19 psychological distress, academic challenges, and academic performance (self-reported predicted GPA). Considered an extension of multiple regression, path analyzes permit the testing of causal links between variables of interests. First, a direct path was estimated between COVID-19 psychological distress and GPA. Next, a composite of academic challenges was entered as a mediator to determine whether COVID-19 impacted students’ academic performance through increasing global academic challenges. Finally, in order to determine the differential impact of various types of academic challenges (i.e., metacognitive, cognitive, behavioral, motivational, and socio-emotional academic challenges), each type of academic challenge was then entered into the model in order to assess for their independent effects over and above the influence of one another. Direct and indirect effects were computed using the MODEL INDIRECT option in Mplus.

## Results

### Descriptives

Means and standard deviations of the variables in the study are provided in Table 1. All of the academic challenges were negatively correlated with self-report GPA (*r*s range from −.15 to −.27; *p*s <.05) and positively correlated with COVID-19 psychological distress (*r*s range from .20 to .42; *p*s <.05). GPA was negatively correlated with COVID-19 distress (r = −.123, *p *< .01).

**Table 1.** Means, Standard Deviations and Correlations for Study Variables.

Variables	Mean	SD	1	2	3	4	5	6	7
1. Predicted GPA	3.41	0.81	1						
2. Motivational	2.06	5.00	−0.227	1					
3. Behavioral	0.81	6.30	−.147**	−.570**	1				
4. Cognitive	1.05	5.82	−.227**	.644**	.588**	1			
5. Socioemotional	4.20	8.44	−.126*	.597**	.515**	.486**	1		
6. Metacognitive	0.33	8.03	−.267**	.587**	.558**	.665**	.613**	1	
7. COVID-19 Distress	18.34	−11.02	−.123**	.252**	.218**	.203**	.418**	.266**	1

Note. All correlations are significant at *p *< .01.

### Mediation Models

**Figure 1.**
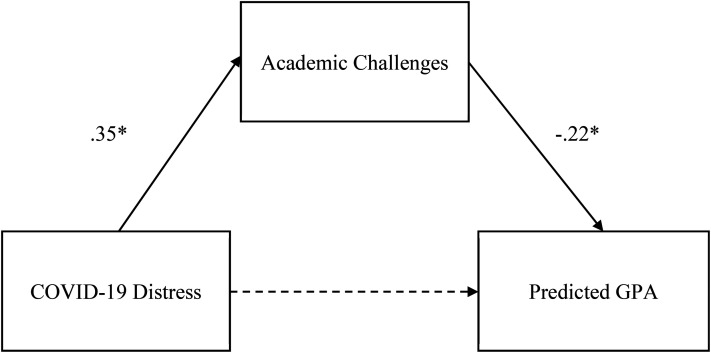
Mediating effect of global academic challenges on the relationship between COVID-19 distress and self-reported predicted GPA. *Note.* Dashed lines represent nonsignificant pathways. *p *<* .001.

A direct path between COVID-19 distress and GPA was first examined before the addition of academic challenge mediators. COVID-19 distress negatively predicted GPA (β = −.12, *p *< .01). A composite of all the academic challenges was subsequently entered into the model in order to assess whether it served as a mediator in these associations. As shown in Figure 1, after including global academic challenges, the association between COVID-19 and GPA was no longer significant (β = −.045; *p* = .327) suggesting the possibility of a full mediation. A significant indirect effect (indirect effect = −.077; *p *< .001) confirmed that the negative association between COVID-19 distress and GPA can be fully explained by academic challenges. Specifically, COVID-19 distress predicted more academic challenges (β = .35, *p* < .001) which in turn predicted lower GPA (β = −.22, *p *< .001).

**Figure 2.**
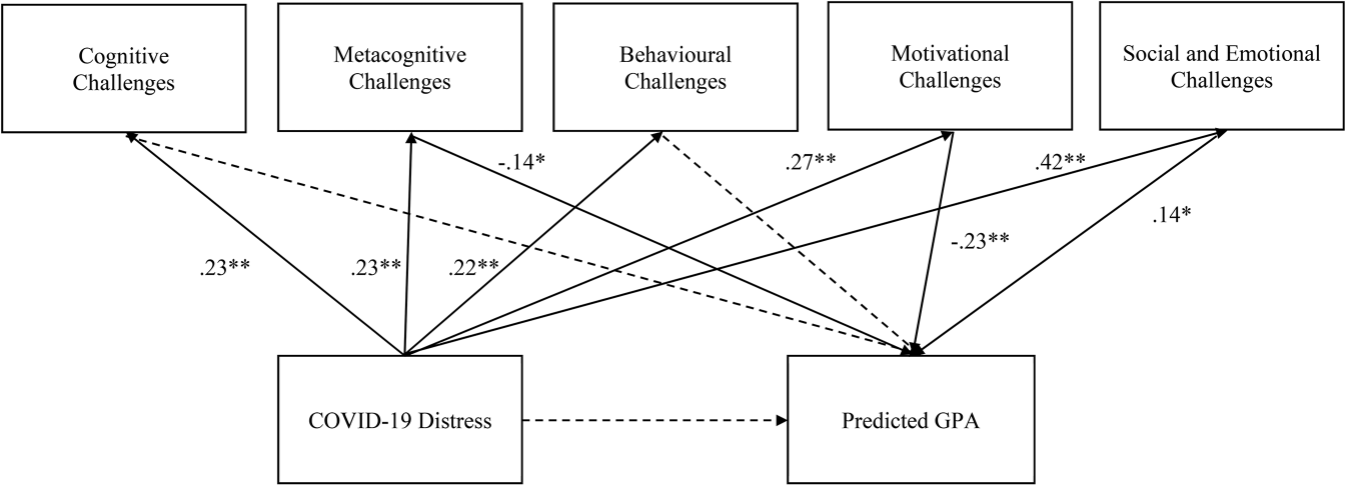
Mediating effect of academic challenges subtypes on the relationship between COVID-19 distress and self-reported predicted GPA. *Note*. Only estimates for significant pathways are presented. All correlations between academic challenges were estimated and significant but are not presented. Dashed lines represent nonsignificant pathways. *p < .05; **p < .001.

Finally, specific academic challenges, cognitive, motivational, social and emotional, behavioral, and metacognitive were analyzed as mediators to determine their unique contribution to COVID-19 distress and GPA. As shown in Figure 2, over and above the influence of the other challenge areas, cognitive and behavioral challenges did not fully mediate the relationship between COVID-19 distress and expected GPA. However, metacognitive, motivational, and social and emotional challenges fully mediated the relationship between COVID-19 distress and expected (indirect effects for metacognitive estimate: −.002, *p* = 0.4; social and emotional estimate: .003, *p* = .02; motivational: −.003, *p* = .002). Specifically greater COVID-19 distress predicted higher levels of metacognitive challenges effect between (β = .23**, *p *< .001) which in turn predicted lower GPA (β = −.14*, *p *< .05). Similarly greater COVID-19 distress predicted more motivational challenges (β = .27**, *p *< .001) which in turn was related to lower expected GPA (β = −.23*, *p *< .05). However, while greater COVID-19 distress predicted more social and emotional challenges (β = .42**, *p *< .001), this increase in turn was related to higher expected GPA (β = .14*, *p *< .05).

## Discussion

The current study examined the impact of psychological distress related to COVID-19 on post-secondary students’ academic performance. Consistent with previous research, greater COVID-19 psychological distress was associated with poorer academic performance as indicated by students’ self-reported predicted GPA (e.g., Cao et al., 2020; Meo et al., 2020). Specifically, our findings demonstrate that students who reported more invasive thoughts related to fears and anxieties about the dangerousness, social and economic consequences of COVID-19, as well as traumatic stress symptoms related to COVID-19 reported lower self-reported GPA. The negative impact of pandemic-related distress on student academic performance is consistent with studies that highlight the adverse effect of psychosocial contextual stressors on students’ GPA (see review by Richardson et al., 2012).

Daily stressors associated with the post-secondary learning environment such as problems related academics, financial concerns, interpersonal challenges, as well as risky health behaviors involving substance use, physical inactivity, poor sleep habits and nutrition are common impediments to students’ academic engagement. The multitude of stressors experienced by post-secondary students place them at greater risk for mental health problems (Monaghan et al., 2021). The pandemic introduces new stressors related to social isolation, academic and financial uncertainty which tax students’ already compromised coping capacities and exacerbates existing academic challenges and hindering performance.

Indeed, our findings reveal that students who reported greater COVID-19 psychological distress reported higher levels of academic challenges. We also found that motivational, metacognitive and social and emotional challenges fully mediated the relationship between COVID-19 distress and GPA suggesting that COVID-19 distress negatively impacts’ students’ academic performance through introducing more of these specific types of challenges. These findings offer a more nuanced understanding of the specific types of academic challenges that are experienced by students who may be differentially by the pandemic.

The finding that pandemic-related psychological stressors affect students’ academic performance by introducing more diverse academic challenges is consistent with self-regulated learning models (Zimmerman, 2000). Self-regulated learning is a dynamic and context-specific process that occurs as learners adjust to a new task and situation at hand. Self-regulated theories highlight processes that are needed for academic success including metacognition, motivation, and strategic action connected to regulating one's cognition, behavior, and socio-emotional factors (Zimmerman, 2008). While academic challenges are normative for students, studying under pandemic conditions introduces unique challenges for students including adjusting to the abrupt switch to remote learning, managing health and financial anxieties, and coping with social isolation that can impair SRL abilities. SRL process models posit that awareness and understanding of one's own learning process or meta-cognitive awareness including regular use of forethought (e.g., strategic planning, goal setting) and self-reflection allow self-regulated learners to more effectively monitor and adapt their learning strategies to shifting contextual changes (Colthorpe et al., 2019; Ward & Butler, 2019; Zimmerman, 2000). Stressors related to the uncertainties of the pandemic coupled with new remote-learning barriers can worsen existing academic challenges particularly among those who already struggle to monitor and adapt their learning strategies effectively to changing demands.

Moreover, the abrupt shift from traditional face-to-face to online learning likely diminishes students’ academic motivations as students face additional self-regulated learning challenges. While online remote learning introduces flexibility for students to “learn anywhere,” it can also pose engagement challenges. When students are physically on campus they have more tools that they can utilize to motivate their studying such as engaging in study groups and better access to support services. Indeed, emerging studies demonstrate that students prefer in-person learning over remote, online learning during the pandemic (Blizak et al., 2020; Langegård et al., 2021). Psychological distress related to the pandemic can also divert motivation as students’ prioritize their physical health over academic engagement especially in geographic locations that may suffer from significant outbreaks. During the pandemic students may place a lower value on their academic tasks thereby diminishing self-regulated motivations and lowering performance.

Psychological distress related to the pandemic impacts students’ academic performance by also increasing metacognitive challenges. Metacognitive strategies as such planning by developing strong task understanding, self-monitoring and self-awareness about what is working promotes self-regulated learning. In pandemic contexts, post-secondary students who are more competent at using self-regulated learning metacognitive strategies are more intrinsically motivated and were less likely to procrastinate than students with lower perceived competence (Pelikan et al., 2021). Metacognitive proficiency is especially important in the remote, online learning environment as planning, time management, and monitoring goal attainment promotes self-discipline and academic motivation. Metacognitive abilities are likely compromised as students struggle to plan, monitor, and regulate their learning while facing intrusive thoughts and anxieties related to the pandemic.

While COVID-distress was associated with more motivational and metacognitive challenges which, in turn, predicted lower self-reported GPA; COVID-distress was associated with more social and emotional challenges but these specific challenges predicted higher self-reported predicted GPA. As post-secondary students report lower mental health during the pandemic (e.g., Blizak et al., 2020; Cao et al., 2020; Meo et al., 2020), it may be that students prioritize academics at the expense of their mental health and relationships which may explain why students who report greater social and emotional academic challenges report higher predicted GPA. Moreover, behavioral and cognitive challenges were not significant mediators over and above meta-cognitive, motivational, and social and emotional challenges. Behavioral engagement such as attending classes may less of an issue in the remote, online learning environment as students are afforded with greater flexibility regarding attendance and assessment options (e.g., open-book assignments and tests). Moreover, while course delivery changed during the pandemic, course content remained the same, thus cognitive challenges may be invariant across the different learning settings.

While the study provides a nuanced understanding of the mechanism between the relationship between COVID-19 distress and student academic success, there are several notable limitations. Participants were sampled predominantly from one academic faculty within one institution which reduces the generalizability of our findings. Moreover, while data were also collected during the height of pandemic in the Fall of 2020 when cases in Canada were rapidly increasing, lack of longitudinal data limits our ability to make inferences on whether students change in their academic experiences and challenges over time. More longitudinal research is needed to discern whether students adapt to their online learning over the course of the pandemic. Studying the relationship between students’ social and emotional challenges and academic performance during less restrictive pandemic conditions (e.g., return to in-person classes, higher proportion of vaccinated students on campus etc.) may provide further insights into how students adapt to their changing learning environments and challenges. Another limitation in the study surrounds the use of self-reports of academic performance (i.e., predicted GPA) which can be subjected to respondent biases. Moreover, students’ psychological state could affect their confidence or ability to predict how they are performing academically particularly among students with poorer metacognitive awareness. Institutional GPA and self-reported GPA would offer additional insights into how pandemic-related academic challenges impact students’ overall academic functioning. Regardless of self-reported predicted GPA, self-report of different types of challenges provides a meaningful assessment of students’ overall academic functioning including their motivation processes and perceptions of how well they monitor, set goals, and adapt effectively to challenges particularly if measures are sensitive to the academic task at hand (McCardle & Hadwin, 2015).

Despite these limitations the study highlights the need to tailor academic intervention efforts to help students manage and cope with their specific academic challenges. Self-regulated learning models posit that academic challenges provide opportunities for students to exercise self-regulatory control by deploying strategies, monitoring progress, and adapting accordingly to the academic challenges they face. During the pandemic, students with fewer SRL skills may be less equipped to cope with the demands of new academic challenges and pandemic stressors as such challenges necessitate more intensive SRL capacities (e.g., metacognitive awareness). Enhancing post-secondary students’ SRL skills through SRL training interventions could help ameliorate academic challenges. Further research is needed to examine the role of self-regulated practices in mitigating the impact of those challenges on academic performance and whether SRL targeted interventions can be leveraged to assist students in overcoming specific academic challenges. Academic intervention programs that help students develop strategic self-regulated learning habits have demonstrated utility in improving academic engagement and performance in both traditional face-to-face (González-Pienda et al., 2014) and online (Tobar et al., 2021) settings. Designing academic interventions that effectively target students’ academic challenges requires understanding the specific problems they face in their academic work. Providing students with a comprehensive assessment of their academic challenges will help to narrow specific self-regulated learning strategies to effectively foster students’ ability to plan, monitor, and manage their thinking, behaviors, and emotion as they engage in their learning (Hadwin & Winne, 2012).
